# Mitigating Global Challenges: Harnessing Green Synthesized Nanomaterials for Sustainable Crop Production Systems

**DOI:** 10.1002/gch2.202300187

**Published:** 2023-12-25

**Authors:** Niranjana Sundararajan, Heena Shabnam Habeebsheriff, Karthikkumar Dhanabalan, Vo Huu Cong, Ling Shing Wong, Ranjithkumar Rajamani, Bablu Kumar Dhar

**Affiliations:** ^1^ Viyen Biotech LLP Coimbatore Tamil Nadu 641031 India; ^2^ Faculty of Natural Resources and Environment Vietnam National University of Agriculture Trau Quy Gia Lam Hanoi 10766 Vietnam; ^3^ Faculty of Health and Life Sciences INTI International University Persiaran Perdana BBN Putra Nilai Nilai Negeri Sembilan 71800 Malaysia; ^4^ Business Administration Division Mahidol University International College Mohidol University Salaaya 73170 Thailand; ^5^ Faculty of Business Administration Daffodil International University Dhaka 1216 Bangladesh

**Keywords:** agriculture, green nanomaterials, nanofertilizer, sustainable crop production

## Abstract

Green nanotechnology, an emerging field, offers economic and social benefits while minimizing environmental impact. Nanoparticles, pivotal in medicine, pharmaceuticals, and agriculture, are now sourced from green plants and microorganisms, overcoming limitations of chemically synthesized ones. In agriculture, these green‐made nanoparticles find use in fertilizers, insecticides, pesticides, and fungicides. Nanofertilizers curtail mineral losses, bolster yields, and foster agricultural progress. Their biological production, preferred for environmental friendliness and high purity, is cost‐effective and efficient. Biosensors aid early disease detection, ensuring food security and sustainable farming by reducing excessive pesticide use. This eco‐friendly approach harnesses natural phytochemicals to boost crop productivity. This review highlights recent strides in green nanotechnology, showcasing how green‐synthesized nanomaterials elevate crop quality, combat plant pathogens, and manage diseases and stress. These advancements pave the way for sustainable crop production systems in the future.

## Graphical abstract

Nanotechnology can help farmers grow more food with less waste, while protecting the environment. Nanofertilizers, nanocomposite biomolecules, and nanobiosensors can improve plant growth, reduce pesticide use, and detect diseases early on.

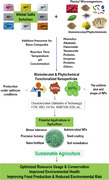

## 1 Introduction

The most advanced industry in the 21st century is nanotechnology. Due to their unique and captivating qualities with a variety of applications, nanoparticles have attracted a lot of attention.^[^ ^1^ ^]^ Nanotechnology is a multidisciplinary, promising area of research that offers numerous prospects in industries like agriculture, electronics, pharmaceuticals, and medicine. Typically, materials with a size between 1 and 100 nm are referred to as nanomaterials. Nanomaterials have unique optical, physical, and biological features because of their small size and large surface area. The creation of nanoparticles can be accomplished through chemical, physical, or biological means.^[^ ^2^, ^3^, ^4^ ^]^ Due to their unique physical and chemical characteristics, nanoparticles (NPs) have been in the spotlight of modern nanotechnology for the past few decades. NPs are useful in a wide range of application areas, including the biomedical and pharmaceutical sectors, wastewater treatment, remediation of environmental pollutants, and food preservation, thanks to several features, including their catalytic, superparamagnetic, antibacterial, and anticancer activities.^[^ ^5^, ^6^, ^7^, ^8^, ^9^, ^10^ ^]^

**Figure 1:**
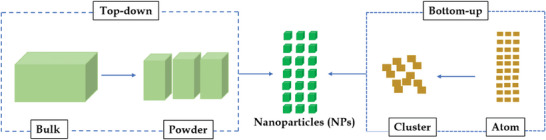
Top‐down and bottom‐up approach of nanoparticles.

The recent focus of nanotechnology has been on the detection and control of plant diseases as well as the creation of nanonutrients to promote plant growth.^[^ ^11^, ^12^, ^13^, ^14^ ^]^ A technique to transport various macro‐ and micronutrients into the soil gradually and in a controlled way, preventing accumulation and polluting of diverse natural resources, is to replace traditional techniques of fertilizer delivery with nanofertilizers. Nanofertilizer innovation necessitates a multidisciplinary approach because it has the ability to outperform conventional fertilizers. In nanofertilizers, nutrients can be delivered as emulsions or nanoparticles, encapsulated by nanomaterials, or coated with a thin protective layer.^[^ ^15^ ^]^ Numerous physical and chemical procedures, including co‐precipitation, microemulsion, laser pyrolysis, sol–gel, and laser ablation techniques, can be used to create nanoparticles.^[^ ^16^, ^17^ ^]^ Green synthesis of NPs is a low‐cost and environmentally friendly manufacturing process that leverages natural biogenic platforms like plant extracts, microbes, or fermented extracts.^[^ ^18^, ^19^, ^20^ ^]^ Additionally, it has the advantages of being quick, safe, energy‐efficient, scalable, and simple to manipulate.^[^ ^19^, ^21^, ^22^ ^]^ Overall, biological approaches provide superior manipulation and control over crystal structure, size, and stabilization of NPs while eliminating the need for toxic and reactive reducing agents that can have a deleterious influence on organisms. It has been widely documented that plant extracts and microorganisms like fungi, yeast, bacteria, and algae can be used to create Fe, Au, Ag, and Zn‐based nanoparticles.^[^ ^23^, ^24^, ^25^, ^26^ ^]^ An increasingly common green synthesis method is the use of bacteria cell‐free supernatant for NP synthesis. Generally, nanomaterial preparation methods can be classified according to assembly mechanisms such as bottom‐up or top‐down techniques (**Figure** **1**). The scope of this review focused on the eco‐friendly preparation of the most important metallic nanoelements using different natural biomasses and bioresources for the sustainable agriculture sector as next‐generation nano‐inputs.

### 1.1 Conventional Nanofertilizers

Conventional nanofertilizers have emerged as a promising solution to address the challenges faced by traditional fertilizers in modern agriculture. These nanoscale materials, typically ranging from 1 to 100 nm in size, offer unique properties that enhance nutrient delivery and uptake efficiency in plants. By encapsulating essential nutrients within nanoparticles, conventional nanofertilizers provide controlled release mechanisms, ensuring a sustained and targeted nutrient supply to crops.^[^ ^27^, ^28^ ^]^ However, despite their potential benefits, conventional nanofertilizers also raise concerns regarding their environmental impact and long‐term sustainability. The widespread application of these nanoparticles may inadvertently lead to the accumulation of toxic residues in soil and water bodies. Additionally, their production processes often involve resource‐intensive techniques that may have adverse ecological consequences.^[^ ^29^, ^30^, ^31^ ^]^

Conventional nanofertilizers, despite their potential in improving crop productivity and nutrient uptake, present certain problems that need to be addressed. First, the high cost of production remains a significant concern. The complex and expensive manufacturing processes involved in synthesizing nanofertilizers make them economically inaccessible for many farmers, particularly those from developing countries. Another issue is the potential environmental impact of conventional nanofertilizers.^[^ ^32^, ^33^, ^34^, ^35^ ^]^ Their increased persistence and mobility in soil can lead to the accumulation of nanoparticles in groundwater, posing risks to aquatic ecosystems. Furthermore, the long‐term effects on soil quality and microbial communities are still not well understood. In addition, concerns over health hazards associated with exposure to nanoparticles have been raised. Inhalation or dermal contact during production or application may lead to respiratory or skin‐related problems for workers or consumers.^[^ ^31^, ^36^, ^37^ ^]^

The use of conventional nanofertilizers in agriculture raises concerns regarding their potential environmental implications. One major concern is the release of nanoparticles into soil and water systems, which may have adverse effects on ecosystems. Studies have shown that these nanoparticles can accumulate in soil and persist for extended periods, leading to potential long‐term environmental contamination. Furthermore, the nanoparticles used in conventional nanofertilizers can also leach into groundwater, posing a risk to drinking water sources.^[^ ^34^, ^37^ ^]^ This contamination may not only affect human health but also disrupt aquatic ecosystems. Additionally, the production and synthesis of conventional nanofertilizers often involve energy‐intensive processes that contribute to greenhouse gas emissions. These emissions contribute to climate change and exacerbate environmental problems. It is crucial to consider the potential environmental impacts associated with conventional nanofertilizers' use.^[^ ^38^ ^]^ In response to the problems associated with conventional nanofertilizers, several alternatives and solutions have been proposed. One potential alternative is the use of organic fertilizers, which are derived from natural sources such as compost or animal manure. These organic fertilizers not only provide essential nutrients but also improve soil structure, water‐holding capacity, and microbial activity. Another solution lies in the development of controlled‐release fertilizers (CRFs).^[^ ^39^, ^40^ ^]^ These CRFs release nutrients gradually over an extended period, ensuring optimal nutrient availability to plants while minimizing nutrient loss through leaching or volatilization. Additionally, nanotechnology‐based encapsulation techniques can be employed to enhance the efficiency of nutrient delivery by protecting them from environmental factors until they reach the target site. Furthermore, precision agriculture techniques like soil testing and remote sensing can be utilized to accurately assess plant nutrient requirements and optimize fertilizer application rates.

## 2 Green Synthesis: A Sustainable Approach

**Figure 2:**
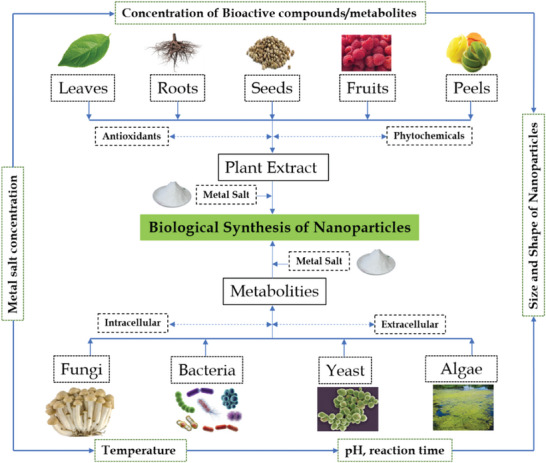
Source of green synthesis of NPs.

Due to its eco‐friendliness and ease of manufacturing compared to other methods, the creation of green nanoparticles generated from plants and microorganisms has gained more interest in the field of green synthesis NPs.^[^ ^41^, ^42^, ^43^, ^44^, ^45^ ^]^ Numerous plant species and microorganisms, such as bacteria, algae, and fungi, are now exploited for NP synthesis in the field of green nanotechnology. Methods, including sonication to dissolve the cell wall, centrifugation to separate biomass, and washing procedures to purify nanoparticles, avoid the requirement for additional cellular recovery of NPs.^[^ ^24^, ^26^ ^]^ By lowering metal ions in aqueous solutions, biocompatible metallic NPs can be made by employing environmentally friendly solvents and reagents, minimizing high‐energy consumption procedures, and using non‐toxic biomolecules such as DNA, proteins, enzymes, carbohydrates, and plant extracts.^[^ ^46^, ^47^ ^]^ If the metabolic condition of the plants is properly addressed, it is possible to synthesize metallic and oxide nanomaterials on an industrial scale using plant tissue culture and downstream processing procedures. It has been discovered that the type of nanoparticles generated by this process differs from plant to plant, depending on the application method, size, and quantity.^[^ ^48^ ^]^ Phytochemicals are more efficient at reducing metal ions compared to fungi and bacteria, indicating that plants are more suitable for nanomaterial production. Fungi, in comparison to bacteria, produce a greater quantity of extracellular enzymes, which play a significant role in nanomaterial synthesis. Fungi are capable of producing nanomaterials with precise dimensions. They can generate a larger quantity of nanomaterials compared to bacteria due to their higher secretion of proteins, which directly contribute to nanomaterial synthesis. Moreover, protein isolates from fungi can be utilized for nanoparticle creation.^[^ ^49^ ^]^ **Figure** **2** shows the source of green synthesis of NPs.

**Figure 3:**
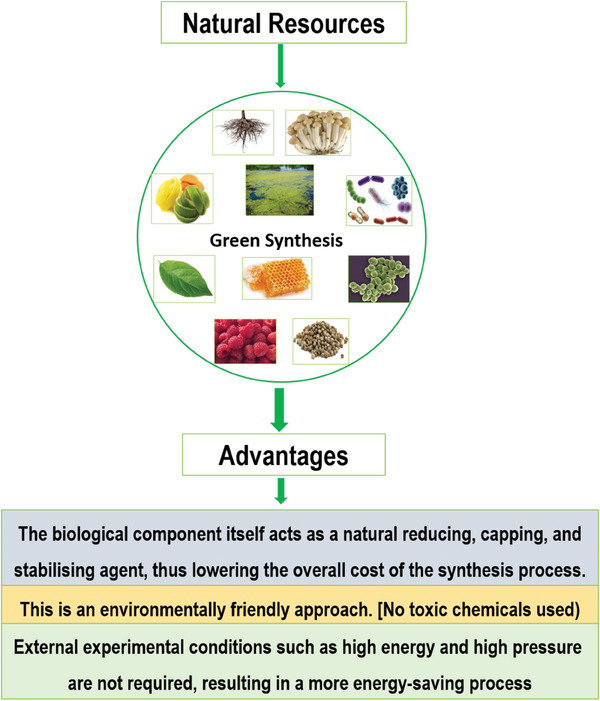
Advantages of green synthesis approach.

Biosynthesized nanoparticles (BioNPs) have demonstrated numerous and advantageous chemical interactions with agricultural systems over the past 10 years, spanning from crop disease management to agricultural production improvement to environmental safety. The plant is demonstrated to be protected from infection by bacteria, fungus, and pests by NPs such as silver (AgNPs), gold (AuNPs), copper (CuNPs), palladium (PdNPs), selenium (SeNPs), zinc oxide (ZnONPs), magnesium oxide (MgONPs), titanium dioxide (TiO_2_NPs), and iron oxide NPs, among others. The bioNPs can act as seed germination and plant growth stimulants in addition to treating crop diseases; increasing crop production.^[^ ^48^ ^]^ **Figure** **3** shows the advantages of the green synthesis approach.

## 3 Impact of Green Synthesis Approach in Improving Crop Quality and Productivity

Fortunately, bioinspired synthesis can enhance the stability and environmental friendliness of nanoparticles (NPs) by adding natural materials to their surfaces. Numerous studies have demonstrated that green synthesis can promote seedling growth and reduce crop phytotoxicity. Therefore, it is crucial to discuss the effects of bioinspired NPs on seed germination, seedling development, and the physiological performance of crops. According to Shaban et al. (2022), zinc oxide nanoparticles (ZnO‐NPs) mediated by pomegranate peel extract reached an optimal concentration of 2 and 12 ppm for both seed germination (90%) and shoot height (6.5 cm), respectively. The greatest root extension (6 cm) was observed at 2 ppm of ZnO‐NPs. All concentrations of ZnO‐NPs significantly improved barley seed germination.^[^ ^50^ ^]^ In another study on the green synthesis of zinc oxide nanoparticles using *Coriandrum sativum* leaves extract by Ukidave et al. (2022), it was shown that a zinc deficiency leads to reduced seed germination rates. Green gram and Turkish gram showed significantly improved growth and development in media treated with ZnO nanoparticles compared to Bengal gram. Zinc deficiency affected protein concentration, chlorophyll levels, and plant development. The use of zinc oxide nanoparticles in conjunction with MS media improved seed germination, plant growth, chlorophyll content, and protein content.^[^ ^51^ ^]^ Sabir et al. (2020) reported increased root and shoot length in maize plants treated with biosynthesized ZnO NPs using *Bacillus* sp. compared to the control, demonstrating a positive impact on plant development. Nano‐priming could be a valuable method for increasing crop productivity.^[^ ^52^ ^]^ Sebastian et al. (2019) produced iron oxide nanoparticles using coconut husk extract rich in phenolics and found that these iron oxide NPs alleviated Cd stress and increased Fe levels in rice plants. This resulted in improved biomass, chlorophyll content, and photosynthetic quantum yield in rice plants. This highlights the importance of iron in respiration, photosynthesis, and chlorophyll biosynthesis.^[^ ^53^ ^]^ Additionally, Karunakaran et al. (2017) investigated the effect of iron oxide nanoparticles on seed germination and antioxidant enzyme levels in flax (*Linum usitatissimum* L. [*L. usitatissimum* L.]) plants. Their findings indicated that an increase in nanoparticle concentration led to increased seedling length (in cm), the average number of seedlings with leaves, and root length (in cm) compared to the control.^[^ ^54^ ^]^ Furthermore, greater levels of iron oxide nanoparticles (Fe_2_O_3_NPs 1000 mg L^−1^) have increased the activity of enzymes, indicated a suppression of ROS formation and promoted plant growth. Besides, the effectiveness of a strategy for producing magnesium hydroxide nanoparticles [Mg(OH)_2_NPs] via green synthesis on *Zea mays* (*Z. mays*)at various concentrations for seed germination, in vitro and in vivo plant growth promotion. The findings showed that plants treated with Mg(OH)_2_NPs had greater Mg levels in their leaves and roots. The fungus *A. niger* extract, which was employed to create Mg(OH)_2_NPs, demonstrated significant promise. The percentage of seed germination was dramatically increased by the biogenic Mg(OH)_2_NPs. Hence it proves that Mg(OH)_2_NPs have the potential to be employed to promote seedling growth and boost seed germination.^[^ ^55^ ^]^ Synthesis of various nanoparticles using bacteria, fungi, plants, and their impacts on plants is listed in **Table** **1**.

**Table 1:** List of green synthesis nanoparticles and their biological applications.

Species	Type of Nanoparticle	Size of nanoparticle [nm]	Biological applications	Reference
*Mentha piperita*	Au	150	Exhibits good antibacterial activity.	[56]
*Bacillus amyloliquefaciens* and *Bacillus subtillis*	Ag	100	*Salmonella, E. Coli, P. Aeruginosa, S. Pyogenes, Staphylococcus aureus* (*S. Aureus*)*, and C. Albicans* have all shown significant antibacterial activity.	[57]
*Klebsiella pneumoniae* (*K. pneumoniae*)	Cu	19–47	Increases plant growth, biomass, and cellular antioxidant content.	[58]
*Trichoderma harzianum*	Ag	51	Shows good antibacterial activity against *S. aureus* and *Klebsiella* in a dose‐dependent way.	[59]
*Rhizopus oryzae*	Ag	7	The outcomes demonstrated that AgNPs exhibited concentration‐dependent antibacterial action.	[60]
Jasmine flower	Ag	40	Exhibit excellent photocatalytic degradation and antimicrobial efficiency.	[61]
*Eriobutria japonica*	ZnO	50	Shows good antibacterial, antioxidant, and photocatalytic activity.	[62]
*Euphorbia esula*	Cu	<32	Shows catalytic activity.	[63]
*Rosa canina*	CuO	15–25	The synthesized nanoparticles were exploited as suitable nanocatalysts for indole *N*‐arylation, aniline, and benzyl amine.	[64]
*Cinnamomum verum*	Fe	20–80	Exhibits better antibacterial, antioxidant, anti‐inflammatory and anti‐diabetic activity against human pathogenic bacteria.	[65]
*Vernonia amygdalina*	MnO_2_	20–22	Used as an excellent reducing agent.	[66]

In order to protect plants from microbial agents and prevent a significant loss of food and agricultural production, it is imperative to identify plant diseases in their early stages. The primary causes of crop loss and decreased agricultural productivity are insect and disease infestations.^[^ ^67^ ^]^ Crop diseases are typically managed using chemical techniques, such as chemical pesticides, biological techniques, such as microbial agents and genetic engineering, and their combinations. On the other hand, due to their outstanding performance in antibacterial activity, NPs are also commercially used for pathogen management. However, the vast applications of conventional NPs are constrained by their possible toxicity and expensive price. As an alternative, bioNPs are more efficient and compatible, offering a plan to control crop diseases.^[^ ^68^ ^]^ For example, *Ustilago tritici* (*U. tritici*) is a seedborne pathogen that causes wheat rust disease. TiO_2_NPs using plant extracts of *Chenopodium quinoa* or *Trianthema portulacastrum* or by a chemical approach and demonstrated that the two types of plant‐derived TiO_2_ had more potent antifungal activity against *U. tritici* than chemical‐derived TiO_2_.^[^ ^69^ ^]^ Rice suffers from bacterial brown strip disease, which is brought on by the soil‐borne bacterial pathogen *Acidovorax oryzae* (*A. oryzae*). Bacterial strains to create MgONPs and chitosan‐magnesium (CS‐Mg) nanocomposites, and they demonstrated that these materials had significant antibacterial activity against *A. oryzae*.^[^ ^70^ ^]^ AgNPs were functionalized using *Sclerocarya birrea* (*S. birrea*) and *Eucomis autumnalis* (*E. autumnalis*) extracts. Two Gram‐negative and two Gram‐positive bacteria showed remarkable antibacterial capabilities. The plant toxicity of the *S. birrea* and *E. autumnalis* AgNPs was minimal or low.^[^ ^71^ ^]^ Antimicrobial activity can also be employed to combat and control fungal diseases. In order to test the antifungal activity of *Melia azedarach* (*M. azedarach*)leaf extract, green manufactured AgNPs from this material. At a dose of 20 ppm, the researchers reported significant effectiveness against *Verticillium dahliae* (*V. dahliae*) in eggplant. Analysis of these data provided information concerning the apparent cytotoxicity, mutagenicity, and genotoxicity of pure AgNPs as well as their anti‐inflammatory and anti‐bacterial activities at different concentrations of synthesized nanoparticles.^[^ ^72^ ^]^ Furthermore, the list of biosynthesized nanoparticles against plant pathogens is listed in **Table** **2**.

**Table 2:** List of green synthesized nanoparticles against plant pathogen.

Pathogen	Nanoparticle used	Plant disease	Action	Reference
*Bipolaris sorokiniana* (*B. sorokiniana*)	AgNPs biosynthesized with *Serratia* sp.	Spot blotch pathogen of wheat.	It helps to Inhibit conidial germination.	[73]
*Xanthomonas oryzae* pv. *oryzae*	ZnO biosynthesized with *Lycopersicon esculentum*.	Bacterial leaf blight diseases of rice.	Showed promising biocontrol agents to combat bacterial leaf blight diseases of rice.	[74]
*Gloeophyllum abietinum*	Green‐synthesized AgNPs extracted with turnip leaf.	Wood‐rotting	It helps in Inhibiting the conidia development.	[75]
*B. sorokiniana*	AgNPs biosynthesized with *Serratia* sp.	Spot blotch pathogen of wheat.	It enhanced the lignification of vascular bundles.	[76]
*V. dahlia*	AgNPs were synthesized using *M. azedarach* leaf extract.	‐	Showed antifungal activity against *V. dahlia* in in vitro and in vivo.	[72]
*Mycenaci tricolor* (*M. tricolor*) and *Colletotrichum* sp.	Green synthesis ZnO NPs with an extract of garlic (*Allium sativum*).	‐	Showed an efficient antifungal capacity, particularly the nanobiohybrids, with ≈97% inhibition in growth of *M. citricolor*, and ≈93% for *Colletotrichum* sp.	[77]
*B. sorokiniana*	Green‐synthesized TiO_2_ NPs with aqueous leaf extract *Moringa oleifera L*.	Spot blotch pathogen of wheat.	Improving the quality and yield of wheat plants by controlling fungal diseases of wheat plants.	[78]
*Colletotrichum* sp.	ZnO NPs from banana peels.	Anthracnose in orchids	High doses of the green synthesized ZnO NPs significantly suppressed the growth of *Colletotrichum* sp. causing Anthracnose symptoms from orchid plants.	[79]
*Alternaria alternata* (*A. alternata*)	Iron nanoparticles synthesized from leaf extracts of *Calotropis procera*.	Black spot disease in leaves.	Significantly inhibited the growth of *A. alternata*.	[80]
*Rhizoctonia solani*	Biologically synthesized Se‐NPs by *Bacillus megaterium*.	Damping off disease and Root rot disease.	Biologically produced Se‐NPs significantly enhanced the amount of carotenoids and total chlorophyll. Se‐NPs functions as a stressor and/or promoter for plants, improving their antioxidative defense systems and promoting plant tolerance.	[81]
*Erwinia amylovora* (*E. amylovora*)	Chromium oxide nanoparticles synthesized from plant extracts of *M. azedarach* and *Artemisia herba‐alba*.	Fire blight disease caused by *E. amylovora*.	Chromium oxide nanoparticles at low concentrations (not more than 100 ppm) can be used as disinfectants *E. amylovora* fire blight bacterium.	[82]
*Fusarium oxysporum* f. sp. *lycopersici*	MgO NPs synthesized from bacterium, *Burkholderia rinojensi*.	Fusarium wilt	MgO NPs caused significant damage in fungal membrane integrity which led to severe fungal morphological alterations and inhibition of biofilm formation.	[83]
*Ralstonia solanacearum* (*R. solanacearum*)	Green synthesized Ag NPs from aqueous leaf extract of *Hypericum perforatum*.	Bacterial wilts in Solanum crops.	Showed antibacterial activity against *R. solanacearum*a major causative agent of Bacterial wilt disease in crops.	[84]
*Botrytis cinerea* and *Colletotrichum gloeosporioides*	Ag NPs synthesized from *Ganoderma applanatum*.	Anthracnose and Gray mold rot disease.	Prevents fungal spreading without affecting the leaf morphology of the plant.	[84]

## 4 Impact of Green Synthesis Approach in Stress Tolerance

The development of stress‐tolerant plants is crucial for the resilience of our planet in the face of climate change and population growth. Plants have evolved various adaptive mechanisms to cope with stress, and one common response is to modify their metabolism, enabling them to better adjust to environmental changes. These metabolic changes involve adjustments at the enzyme and gene levels, such as increasing or decreasing the activity of specific enzymes or activating and deactivating certain genes. These adjustments are triggered by different types, frequencies, and intensities of stress events, including drought, salinity, aridity, and cold. Green synthesis mediated nanomaterials approaches offer a promising strategy for enhancing stress tolerance in plants through biochemical, molecular, and cellular interventions. They provide insights into the role of the plant's photosynthetic apparatus in stress tolerance and help in understanding the mechanisms observed at the cellular level. This knowledge opens up new possibilities for designing biomimetic materials with improved performance against environmental stresses, which could have applications in human health. Additionally, these nanomaterials possess unique properties that make them valuable in applications requiring high standards, such as gas storage and separation, catalysis, sensing, and chemical/biochemical separations. They also show potential for use as sacrificial materials in environmental cleanup and wastewater treatment.

Silver nanoparticles (AgNPs) have gained increasing popularity in agriculture due to their impact on stress tolerance. They have been explored in various forms for their potential role in protecting against abiotic stress. AgNPs have been shown to improve crop stress tolerance by addressing nutritional deficiencies, enhancing enzymatic activities, and promoting the adherence of plant growth‐promoting bacteria to plant roots under abiotic stresses. These findings highlight the potential of AgNPs as a valuable tool for enhancing crop resilience to stress. AgNPs have a positive effect on combating drought stress in germination rate of lentils and germination parentage was highest at zero water potential by enhancing phenotypic traits of the plant.^[^ ^85^, ^86^ ^]^ It is stated that biologically synthesized nanoparticles such as TiO_2_NPs^[^ ^87^ ^]^ and AgNPs^[^ ^88^ ^]^ have non‐toxic, reduce oxidative stress in plants with improved antioxidant defense mechanisms under abiotic stress conditions. The nanoparticles act as capping agents, making them biocompatible and exhibits optical and photocatalytic properties, strong oxidation potential and stability. Iron levels in the soil environment plays a key role in the plant metabolism and several studies demonstrate the effect of Iron (Fe) based nanoparticles, for example, Fe_3_O_4_NPs at low concentrations favor plant development (increment in root length and photosynthetic rate) in *Fragaria × ananassa* plantlets, through their absorption and enhanced stability when compared to the control plants under drought stress.^[^ ^89^ ^]^ Another study suggests a rise in antioxidant enzyme activities, their biomass concentration, enhanced nutrient uptake ability, and photosynthetic efficacy of Fe_3_O_4_NPs‐treated *Oryza sativa* (*O. sativa*) plants during drought stress conditions.^[^ ^90^ ^]^ Therefore, these studies evident that application of iron based metal oxide nanoparticles in agriculture presents promising results and could be considered as eco‐friendly plant growth enhancers. Furthermore, the list of biosynthesized nanoparticles to enhance stress tolerance is listed in **Table** **3**.

**Table 3:** List of Green synthesized nanoparticles and their application in stress tolerance.

Species	Abiotic Stress	Green synthesized NPs Treatment	Effects	References
Lentil (*Lens culinaris)*	Drought stress	Ag	Enhanced shoot length, germination rate, germination percentage, fresh, and dry weight.	[86]
*Triticum aestivum* L. (*T. aestivum* L.)	Salt stress	Ag	Upregulated anti‐oxidative enzymes and protective oxidative damage during saline stress.	[91]
*T. aestivum* L.	Drought stress	TiO_2_	Improved plant height, ear weight and number, seed number, increased harvest index, and biomass. Also, increased gluten and starch contents.	[92]
*Phaseolus vulgaris L*.	Chilling temperatures	Ag	Improved chilling stress tolerance resulting in increased photosynthesis, seed germination, fresh, and dry weight.	[93]
Saffron (*Crocus sativus)*	Flooding stress	Ag	Enhanced root and leaves dry and fresh weight.	[94]
*T. aestivum* L.	Heat stress	Ag	Improved chlorophyll content.	[88]
*T. aestivum* L.	Drought stress	TiO_2_	Increase in photosynthetic pigments, seed germination rate, fresh, and dry weight.	[95]
*Lalleman tiaiberica*	Drought stress	TiO_2_	Increase in protective antioxidant enzymes.	[96]
*L. usitatissimum* L.	Drought stress	TiO_2_	Increase in photosynthetic pigments, seed germination rate, fresh, and dry weight.	[97]
*Ocimum basilicum L*.	Drought stress	TiO_2_	Enhanced germination rate, germination percentage, fresh, and dry weight.	[98]
*Vigna radiata*	Drought stress	ZnO	Enhanced root biomass.	[99]
*Z. mays*	Drought stress	ZnO	Increased photosynthetic activity and chlorophyll content in maize seedlings. Improved water use efficiency. Enhanced the glycolysis metabolism and biosynthesis of starch and sucrose in leaves under drought stress.	[100]
*Sorghum bicolor* (*S. bicolor*)	Drought stress	ZnO	Improved nutrient uptake, enhanced growth, and yield.	[101]
*S. bicolor*	Salt stress	ZnO	Improved osmoprotectant efficiency, membrane stability, and maintains cellular homeostasis.	[102]
*T. aestivum* L.	Drought stress	ZnO	Decreased oxidative stress due to Increased antioxidant enzyme efficacy and indicators in wheat leaves.	[103]
*Moringa peregrina*	Drought stress	ZnO	Enhanced osmoprotectant content and antioxidant activity thereby, oxidative stress is reduced.	[104]
*Fragaria* × *ananassa*	Drought stress	Fe_3_O_4_	Improved the relative water content. Increased membrane stability, plant growth, and photosynthetic pigments.	[105]
*O. sativa*	Drought stress	Fe_3_O_4_	Increase in biomass, antioxidant enzyme activities, photosynthetic efficiency, and nutrient uptake.	[70]
*Fragaria* × *ananassa*	Drought stress	Fe_3_O_4_	Increases in photosynthetic rate and root length.	[89]
*T. aestivum* L.	Heavy metal stress	CuNPs	CuNPs‐treated wheat plants significantly increased the root length and shoot length of under the Cr stress in wheat plants.	[58]

## 5 Green Synthesis Approach in Soil Remediation

The green synthesis nanoparticle approach is being employed for the remediation of contaminated soil in agriculture. These nanoparticles have proven effective in removing contaminants from the soil and restoring its quality. Nanomaterials, particularly those produced through green synthesis approaches, show great promise in environmental remediation due to their cost‐effectiveness and eco‐friendliness. Soil contamination is a significant issue in agricultural regions, leading to substantial agronomic losses. The contamination often arises from the use of harmful pesticides and industrial effluent dyes. This contamination negatively impacts plant growth and crop yields by disrupting the soil's nutrient balance, hindering plants' access to essential nutrients, and ultimately causing wilting and death. In addition to affecting plant growth, soil contamination poses risks to human health as contaminants are absorbed by plants, and certain industrial heavy metals can leach into groundwater, rendering it unsafe for consumption. The biological synthesis of nanomaterials offers a solution to mitigate the harmful effects of soil contamination by providing a protective barrier against contaminants. This barrier safeguards plants, allowing them to thrive in contaminated soils and promoting plant growth and crop yields.

Green synthesized nanomaterials can also facilitate the breakdown of contaminants in the soil, facilitating nutrient absorption and growth in plants, while simultaneously reducing overall soil contamination levels.

Scientists have engineered bioinspired metal‐based nanoparticles such as AgNPs, ZnNPs, AuNPs, FeNPs, CdNPs, PdNPs, TiO2 NPs, and CuNPs, to combat highly toxic pollutants in metal‐contaminated soils. Additionally, these nanoparticles can serve as biosensing materials, indicating the presence of heavy metal contamination in the environment. **Table** **4** illustrates some of the bioinspired nanoparticles used in environmental remediation in agriculture.

**Table 4:** List of Green synthesized nanoparticles and their application in environmental remediation.

Source	Bioinspired derived Nanoparticles	Effect	Reference
Leaf extracts of *Azadirachta indica* and *Mangifera indica*	Ag NPs	Effective detection of lead, zinc, and mercury heavy metal contamination in water system.	[106]
Leaf extracts of *Centella asiatica (L.)*	CuO NPs	Significant degradation of methyl orange dye.	[107]
Dried fruit extracts of *Carissa edulis*	ZnO NPs	Exhibits good reducing property and efficient photocatalytic degradation of Congo red dye.	[108]
Aqueous leaf extract of *Catharanthus roseus* (*C. roseus*)	Pd NPs	Pd NPs effectively degrades the phenol red dye at an optimum pH of 8.0.	[109]
Rice husk	Graphene quantum dots	Acts as effective nonabsorbent. Exhibits high thermal stability property and effectives binds to lead (II) and lanthanum (III) ions and remediates the metal contamination.	[110]
*Alpinia calcarata Ocimum sanctum Cymbopogon citratus*	Nanoscale zero valent iron (n ZVI)	Effectively remediates endosulfan pesticide by sequential dehalogenation and hydrogenolysis.	[111]
*Pteris cretica*	Nanobiosensors detect Arsenic heavy metal contamination.	The roots of the plants were able to tolerate and hyper accumulate exceptionally high level of arsenic for selective arsenite detection.	[112]
*K. pneumoniae*	CuNPs	Effectively reduced the toxicity of chromium (VI)‐contaminated soils to wheat plants. In addition, there was an increase in root and shoot length of the plant.	[58]
*C. roseus*	Ag, Cu	Efficient removal of chromium and cadmium metals. Also exhibits antibacterial activity against *S. aureus*.	[113]
*Vitex agnus‐castus* fruits	SnO_2_	The rhodamine B (RhB) dye degradation efficiency of SnO_2_ nanoparticles stated enhanced photocatalytic activity. Also, these NPs can be used as an adsorbent for removal of heavy‐metal ions of Co^+2^. The removal efficacy was obtained to be higher than 94%.	[114]

## 6 Prospects, Challenges, and Future Perspective of Green Synthesis Approach

**Figure 4:**
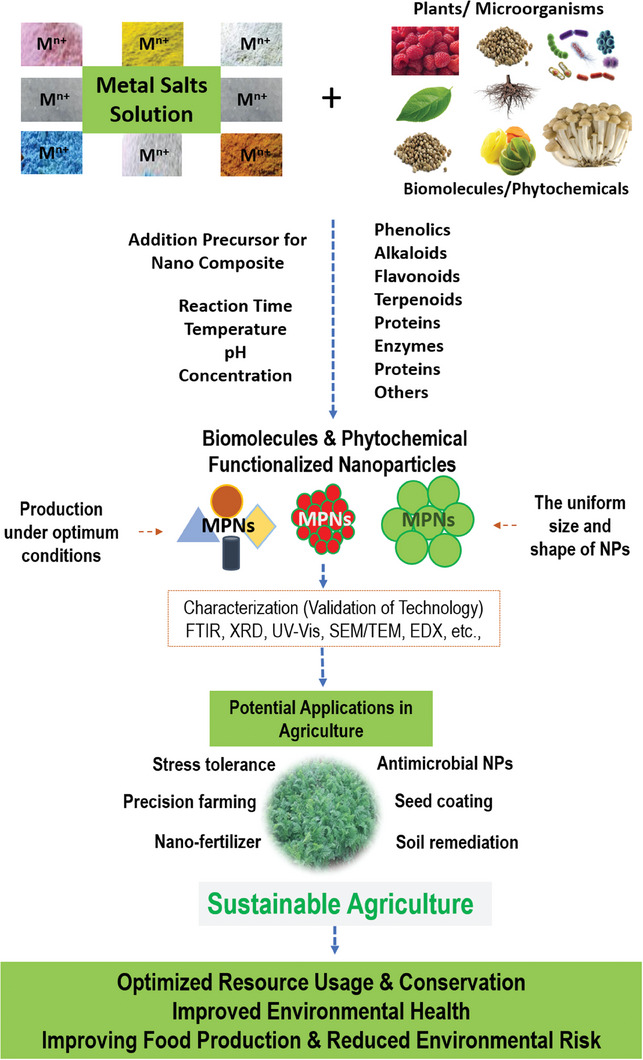
Ecofriendly NPs for sustainable agriculture.

The concept of biologically creating nanoparticles is still in its early stages. Green synthesis technology holds great promise due to its potential for simplicity, effectiveness, cleanliness, non‐toxicity, and environmental acceptability in the synthesis of metallic nanoparticles (MtNPs). MtNPs can be efficiently biosynthesized using various microbes and plant extracts. However, further extensive research is needed to fully understand and enhance the wide range of applications and transformative potential of biosynthesized NPs. Significant progress has been made in the past decade regarding the production of biogenic NPs and the identification of their diverse range of suitable uses (**Figure** **4**). The utilization of MtNPs in agriculture plays a significant role in mitigating the negative effects of excessive pesticide and artificial fertilizer use, which is crucial for improving agricultural productivity amidst challenges posed by population growth and global climate change.^[^ ^115^ ^]^ The application and controlled release of insecticides and fertilizers through various nanoformulations contribute to the prevention of environmental damage. However, there are certain limitations, such as the lengthy synthesis process, labor‐intensive procedures, and challenges in controlling morphology. So far, the biological synthesis of metallic nanoparticles has primarily been conducted in laboratory settings. Achieving large‐scale production requires optimization at an industrial level. These “bio‐nano‐factories” have the potential to produce stable nanoparticles with well‐defined sizes, compositions, and morphologies, given the optimal conditions and appropriate microorganisms. The commercialization of these methods will lead to the development of a non‐toxic biological system capable of generating metallic nanoparticles, marking another significant stride toward sustainable development.

There is a critical need for further research into biosynthetic pathways as they hold the potential to serve as a foundational basis for controlling the characteristics of bioNPs. It is imperative to continue exploring methods for precise regulation of bioNPs properties to enable their specific applications. Furthermore, it is crucial to elucidate how bioNPs influence plant physiology and their molecular‐level absorption by plants as micronutrients. Addressing these challenges can be achieved by manipulating the biosynthesis system at the genomic and proteomic levels and employing genetic engineering techniques. Mechanistic understanding of the proteins and enzyme systems involved in the production of nanoparticles should be a focus of future research, as it remains unclear how biologically created nanoparticles function. Additionally, comprehensive research comparing the characteristics of manufactured nanoparticles to their chemical counterparts is necessary. This presents a significant opportunity to investigate potential bioNPs synthesis processes and their applications in the agricultural industry. Overcoming these barriers holds immense potential for bioinspired synthesis of NPs to greatly benefit future generations in advancing sustainable agriculture. This review provides a detailed analysis of contemporary biosynthetic methods for bioNPs and their role in enhancing crop quality and quantity. Furthermore, the research delves into the effects of the green synthesis strategy on plant pathogen defense, stress tolerance, soil remediation, and environmental remediation within the context of sustainable agriculture.

## 7 Green Biosensors for Plant Disease Detection

Plant diseases pose a significant threat to global agriculture, leading to severe economic losses and food scarcity. Early detection and monitoring of these diseases are crucial for effective disease management and prevention, ultimately reducing crop losses. Traditional methods for disease detection are often time‐consuming, labor‐intensive, and require specialized expertise. However, the emergence of green biosensors has revolutionized the field of plant disease diagnostics. Green biosensors have emerged as a promising solution for early disease detection in plants. These biosensors utilize living organisms or their components such as enzymes or antibodies, to identify specific pathogens or disease indicators accurately^[^ ^116^, ^117^, ^118^ ^]^ to detect specific pathogens or pathogen‐induced changes in plants.^[^ ^119^, ^120^, ^121^, ^122^ ^]^ Green biosensors offer several advantages over conventional methods, including rapid detection, high sensitivity, cost‐effectiveness, and portability. Biosensors have emerged as valuable tools for plant disease detection due to their sensitivity, specificity, and rapid response. These green biosensors employ biological components, such as antibodies or nucleic acids, coupled with transducers to convert the recognition event into a measurable signal. The principles underlying biosensor functioning involve the specific binding of target molecules present in plants infected by pathogens. Upon interaction with the target molecule, a signal is generated by the transducer, which is then quantified and analyzed.^[^ ^123^, ^124^, ^125^, ^126^ ^]^ The sensitivity of biosensors allows for early disease detection even before visible symptoms manifest in plants. Furthermore, biosensors can be tailored for different plant diseases by selecting suitable biological recognition elements, making them versatile tools for effective disease management in agriculture.

## 8 Types of Green Biosensors Used in Plant Disease Detection

Green biosensors for plant disease detection are a crucial tool in agricultural and environmental monitoring. Various types of biosensors have been developed, each with its specific mechanism and advantages. One type is the optical biosensor, which utilizes light absorption or emission to detect disease‐related changes in plants. Another type is the electrochemical biosensor, which measures electrical signals resulting from biochemical reactions associated with plant diseases.^[^ ^127^, ^128^ ^]^ Additionally, nanobiosensors have gained prominence due to their high sensitivity and small size, enabling real‐time monitoring at the cellular level. Moreover, genetic‐based biosensors utilize gene expression patterns or DNA sequences to identify specific pathogens or disease markers in plants. By harnessing these diverse types of green biosensors, researchers can accurately and rapidly detect plant diseases, aiding in timely interventions for crop protection and sustainable agriculture practices.^[^ ^129^, ^130^ ^]^ Recent advancements in green biosensor technology have revolutionized plant disease detection by improving sensitivity, selectivity, and portability. Some researchers incorporate nanomaterials, such as carbon nanotubes and graphene, into biosensor platforms to enhance signal transduction and improve detection limits.^[199,^ ^124^, ^131^, ^132^ ^]^ Additionally, the integration of smartphone‐based technologies has enabled real‐time monitoring and remote data analysis, making these biosensors accessible even in resource‐limited settings.^[^ ^122^ ^]^

## 9 Future Directions and Potential Impact of Green Biosensors on Agriculture

Green biosensors offer several advantages in the field of plant disease detection. First, they provide a rapid and sensitive method for early disease detection, enabling timely intervention and preventing extensive crop losses. Additionally, these biosensors are cost‐effective compared to traditional diagnostic techniques, making them suitable for large‐scale implementation in agriculture. Furthermore, green biosensors are user‐friendly and do not require specialized training or equipment, allowing farmers with limited resources to benefit from their use.^[^ ^119^, ^123^, ^133^ ^]^ However, green biosensors also have certain limitations. One major drawback is their specificity since some biosensors may not accurately differentiate between different pathogens or strains of the same pathogen. Another limitation is the need for continuous monitoring as plant diseases can rapidly evolve and new strains may emerge over time.^[^ ^124^, ^134^, ^135^ ^]^ In addition to this, two more case studies indicate successful applications of green biosensors in plant disease dentition. Tomato Leaf Mold: In a recent study, researchers successfully utilized green biosensors to detect and monitor the presence of *Cladosporium fulvum*, the causal agent of tomato leaf mold. The biosensor, incorporating fluorescent nanoparticles, exhibited high sensitivity and specificity in detecting the pathogen at early stages. This allowed for timely disease management strategies, minimizing yield losses. Citrus Canker another case study focused on citrus canker detection using green biosensors demonstrated promising results.^[^ ^121^, ^123^, ^132^, ^136^ ^]^ By harnessing the plant's own defense response mechanisms, researchers engineered a biosensor that detected *Xanthomonas citri* subsp. *citri*, the bacterium causing citrus canker infection.^[^ ^118^, ^128^ ^]^ The development of green biosensors for plant disease detection holds immense potential for revolutionizing agriculture practices. As researchers continue to advance this technology, several future directions can be envisioned. First, there is a need to enhance the sensitivity and specificity of these biosensors to accurately detect a wide range of plant pathogens. Additionally, efforts should be focused on miniaturizing these devices for easy integration into existing agricultural systems.^[^ ^126^, ^128^ ^]^ The incorporation of wireless communication capabilities would enable real‐time monitoring and data analysis, facilitating timely disease management decisions. Furthermore, the integration of artificial intelligence algorithms can assist in the interpretation of complex data patterns, enabling rapid identification and response to emerging plant diseases.^[^ ^137^ ^]^

## 10 Conclusion

This research sheds light on the prospects, challenges, and future outlook of the green synthesis approach in sustainable crop production systems. By advancing our understanding in this field and addressing the remaining obstacles, we can pave the way for a more environmentally friendly and efficient agricultural industry that actively contributes to global sustainability. The research conducted on green‐synthesized nanomaterials for sustainable crop production systems has revealed the immense potential of this method in agriculture. Green nanotechnology, which boasts minimal environmental impact and substantial economic benefits, offers a promising solution to the limitations associated with chemically synthesized nanoparticles. By harnessing the power of green plants and microorganisms as sources for nanoparticle production, the agricultural sector can take advantage of various applications such as fertilizers, insecticides, pesticides, and fungicides.

Notably, nanofertilizers have demonstrated their efficacy in minimizing mineral losses during fertilization, enhancing yield through improved mineral management, and promoting overall agricultural development. The biological approach to nanoparticle production has proven to be environmentally friendly, cost‐effective, and capable of generating nanoparticles with high purity. By highlighting the role of natural phytochemicals in augmenting crop productivity, this research provides a comprehensive overview of the advancements in green nanotechnology and the utilization of green synthesis nanomaterials.

Although the field of biologically synthesized nanoparticles is still in its early stages, significant progress has been achieved in the past decade. The incorporation of these nanoparticles in agriculture presents solutions to the challenges posed by excessive pesticide usage and artificial fertilizers, thus helping to mitigate the adverse environmental effects. However, several obstacles must be addressed, including the optimization of large‐scale production processes, control over nanoparticle morphology, and the elucidation of the mechanisms and absorption of bioNPs by plants. Future research endeavors should focus on exploring biosynthetic pathways, precise regulation of bioNPs characteristics, and investigating the proteins and enzyme systems involved in their production. Furthermore, it is crucial to comprehensively understand the properties of bioNPs in comparison to their chemical counterparts. Overcoming these barriers presents a significant opportunity to fully unlock the potential of bioinspired synthesis of nanoparticles, ultimately contributing to sustainable agriculture and benefiting future generations.

## Conflict of Interest

The authors declare no conflict of interest.

## Author Contributions

N.S., H.S.H., and K.D.: data collection and draft of manuscript. B.K.D., V.H.C., T.T.T.H., and R.R.: outlining and rewriting. B.K.D., L.S.W., and S.D.: editing and final draft of manuscript. All authors contributed to the article and approved the submitted version.
